# Dimethyl Fumarate Ameliorates Doxorubicin-Induced Cardiotoxicity By Activating the Nrf2 Pathway

**DOI:** 10.3389/fphar.2022.872057

**Published:** 2022-04-26

**Authors:** Xiaoliang Hu, Cheng Li, Qian Wang, Zhixing Wei, Taizhong Chen, Yuepeng Wang, Yigang Li

**Affiliations:** Department of Cardiology, Xinhua Hospital Affiliated to Shanghai Jiao Tong University School of Medicine, Shanghai, China

**Keywords:** dimethyl fumarate, doxorubicin, oxidative stress, apoptosis, Nrf2 pathway

## Abstract

Doxorubicin (DOX) is limited in clinical application because of its cardiotoxicity. Oxidative stress and apoptosis are crucial in DOX-induced cardiac injury. Dimethyl fumarate (DMF) is an FDA-approved oral drug with powerful effects to reduce oxidative stress and apoptosis through the Nrf2 pathway. This study was aimed to determine whether DMF can protect against DOX-induced cardiac injury. We used both neonatal rat cardiomyocytes (NRCMs) *in vitro* and DOX-induced cardiac toxicity *in vivo* to explore the effects of DMF. The results showed that DMF significantly improved cell viability and morphology in NRCMs. In addition, DMF alleviated DOX-induced cardiac injury in rats, as evidenced by decreased CK-MB, LDH levels, improved survival rates, cardiac function, and pathological changes. Moreover, DMF significantly inhibited cardiac oxidative stress by reducing MDA levels and increasing GSH, SOD, and GSH-px levels. And DMF also inhibited DOX-induced cardiac apoptosis by modulating Bax, Bcl-2 and cleaved caspase-3 expression. Moreover, DMF exerted its protective effects against DOX by promoting Nrf2 nuclear translocation, which activated its downstream antioxidant gene Hmox1. Silencing of Nrf2 attenuated the protective effects of DMF in NRCMs as manifested by increased intracellular oxidative stress, elevated apoptosis levels, and decreased cell viability. In addition, DMF showed no protective effects on the viability of DOX-treated tumor cells, which suggested that DMF does not interfere with the antitumor effect of DOX *in vitro*. In conclusion, our data confirmed that DMF alleviated DOX-induced cardiotoxicity by regulating oxidative stress and apoptosis through the Nrf2 pathway. DMF may serve as a new candidate to alleviate DOX-related cardiotoxicity in the future.

## Introduction

Doxorubicin (DOX) is isolated from a mutated strain of Streptomyces peucetius var. caesius and is widely used in clinical for multiple malignant tumor treatment (Carvalho et al., 2009; Damiani et al., 2016). It is one of the most established and commonly used antineoplastic agents in various cancers, including pediatric cancer, leukemia, breast cancer, etc. Unfortunately, this drug can cause cardiotoxicity, including arrhythmia, hypotension, heart failure, and even late-onset cardiomyopathy (Nebigil and Desaubry, 2018). The incidence of heart failure will climb to 48% once the accumulation dose of DOX reaches 700 mg/m2 (Li and Hill, 2014). Thus, the application of DOX is limited despite its powerful anti-tumor characteristic. Currently, the only FDA-approved cardioprotective drug for DOX is dexrazoxane, which does not interfere with DOX activity (Reichardt et al., 2018). The hydrolysis products of dexrazoxane could prevent the generation of cardiotoxic reactive oxygen species (ROS) by chelating intracellular iron. However, hematological toxicity such as severe leucopenia was more common in patients with dexrazoxane, which may interfere with chemotherapy (Wiseman and Spencer, 1998). The mechanisms of DOX-induced myocardial injury include oxidative stress, lipid peroxidation, DNA damage, mitochondrial injury, apoptosis, and autophagy disorder (Rawat et al., 2021). Among them, oxidative stress and apoptosis-mediated cardiomyocytes death are the leading cause of cytotoxicity (Octavia et al., 2012). Briefly, DOX produces massive ROS, which induces mitochondrial dysfunction and cardiomyocyte apoptosis (Kashfi et al., 1990; Schlame et al., 2000). Therefore, targeting oxidative stress and apoptosis should be effective against DOX-induced cardiotoxicity.

Dimethyl fumarate (DMF), known as Tecfidera, is an FDA-approved drug for severe psoriasis and relapsing multiple sclerosis (MS) since 1994 (Xu et al., 2015). DMF is a fumaric acid, which can mainly be hydrolyzed by esterase into monomethyl fumarate (MMF) with a half-life of 1 h. Both DMF and MMF exert similar pharmacological effects in several pathological conditions. Evidence suggests that DMF mainly exerts protective effects by activating the nuclear factor erythroid 2 (Nrf2) antioxidant pathway (Scannevin et al., 2012). Nrf2 is an important transcription factor responsible for regulating the redox balance within the cell. Under normal conditions, Nrf2 remains inactive in the cytoplasm due to its binding to the Keap1 protein and secondary ubiquitination degradation. However, DMF can oxidize the sulfhydryl groups of Keap1, thereby separating Keap1 from Nrf2. Then Nrf2 enters the nucleus to activate various powerful antioxidant genes, including heme oxygenase-1 (Hmox1), NAD (P) H-quinone dehydrogenase 1 (NQO-1), and glutathione Peptide-S-transferase 1 (GST-1) (Ma, 2013). Besides, DMF has a strong anti-inflammatory effect by inhibiting NF-κB activity and many inflammatory cytokines expressions such as iNOS, TNF-a, and IL6 (Wierinckx et al., 2005; Meili-Butz et al., 2008; Wilms et al., 2010; Scannevin et al., 2012). Given the powerful effects of regulating oxidative stress and inflammation, DMF has been already shown benefits in treating several diseases such as COPD (Cattani-Cavalieri et al., 2020), IBD (Li et al., 2020) and recently novel coronavirus (COVID-19) infection (Olagnier et al., 2020).

As a powerful drug to activate Nrf2, DMF has shown protective effects in several cardiac pathological models such as myocardial infarction, ischemia-reperfusion injury, and sepsis-induced cardiac dysfunction (Meili-Butz et al., 2008; Giustina et al., 2018; Mouton et al., 2021). Here we hypothesize that DMF might also protect against DOX-induced myocardial injury. However, this has not previously been reported. The objectives of the current study were to investigate whether DMF can protect against DOX-induced cardiac damage.

## Materials and Methods

### Chemicals and Materials

DOX was obtained from Selleck. DMF was purchased from Selleck, which was dissolved in 0.8% Carboxymethyl cellulose (CMC) for *in vivo* tests and 0.1% dimethylsulfoxide (DMSO) for *in vitro* experiments. The nuclear and cytoplasmic protein extraction kits were purchased from KEYGEN Biotech. Co., Ltd. (Nanjing, China). Bicinchoninic acid (BCA) protein assay kit and cell lysis buffer kit were obtained from Beyotime Institute of Biotechnology (Jiangsu, China).

### Primary (Neonatal Rat Cardiomyocytes) NRCMs Culture

NRCMs were isolated from the ventricles of 1- to 3-day-old neonatal Sprague–Dawley (SD) rats as previously described (Liu et al., 2014). Briefly, neonatal rat hearts were minced into 1-mm^3^ pieces and were digested with 0.125% trypsin and 0.1% collagenase type I. Tow hours differential attachment culture was performed to separate cardiac fibroblasts from cardiomyocytes. Then NRCMs were cultured in medium with 5-BrdU. Through this method, the purity of cardiomyocytes could reach more than 90%. After incubation for 24 h, 90% of cardiomyocytes exhibited spontaneous pulsing, which indicated good viability.

### Animals

Male-SD rats weighing 230–250 g (8-weeks old) were obtained from the Shanghai Jihui Laboratory Animal Care Co., Ltd (Shanghai, China) and maintained under SPF conditions in a controlled environment of 20–22 °C, with a 12/12 h light/dark cycle and 50–70% humidity, and food and water provided ad libitum. Rats were randomly divided into five groups: control groups, DOX-treated groups, solvent control groups, and DMF-treated groups. DMF was dissolved in 0.8% CMC and administered to rats by oral gavage, with CMC as solvent control. Based on preliminary data and previous studies, the rats were treated with DMF at a total daily dose of 40 mg/kg/d and 80 mg/kg/d twice a day (Li et al., 2018; Motterlini et al., 2019). The rats in DOX-treated groups were intraperitoneally injected with DOX (15 mg/kg diluted with 0.9% saline). The rats in solvent control groups and DMF-treated groups were pretreated with 0.8% CMC or DMF a week prior to DOX treatment (15 mg/kg diluted with 0.9% saline, intraperitoneally) and maintained until the end of the experiment. Six days after DOX treatment, all rats were sacrificed. The serum samples were obtained from blood by centrifugation (3000 r/min, 4°C) for 10 min, and the heart tissues were removed for further testing. All animal experiments were approved by the Institutional Review and Ethics Board of Shanghai Xinhua Hospital, Shanghai Jiao Tong University School of Medicine.

### Echocardiography Analysis

Rats were anesthetized with 1% isoflurane inhalation and placed on a heated pad to maintain 37°C body temperature. The ejection fraction (EF) and fractional shortening (FS) were measured from M-mode images of echocardiography (Vivid 7; GE Medical, Milwaukee, WI, United States) with a 15 MHz transducer.

### Cell Viability Evaluation

Cell counting kit 8 (CCK8) assay was performed according to the manufacturer’s instructions. Briefly, after the cells were exposed to various treatments, CCK-8 (10 µl) was added to each well of the 96-well plate, and the plate was incubated for 4 h at 37°C. Cell viability was calculated by absorbance measurements at 450 nm using a Synergy H1 Multi-Mode Reader (BioTek, Winooski, VT, United States).

### Hoechst 33258 Staining

Cells were seeded in the 24-well plates and were incubated with different interventions. At the end of the incubation period, cells were fixed, washed with PBS three times, and stained with Hoechst 33258 staining solution (Beyotime, Shang Hai, China) for 5 min at room temperature and observed by fluorescence microscope (OLYMPUS, Tokyo, Japan). Fragmented or condensed nuclei were considered apoptotic cells.

### ROS Testing

At the end of different interventions, DCFH-DA (10 μM) was added in the well for 20 min induction at 37°C after removing the medium. The samples were observed using fluorescence microscopy (Olympus, Tokyo, Japan).

### Immunofluorescence Staining

NRCMs were stained with antibodies against α-actinin (A5044,1:100) and Nrf2 (ab137500,1:100), heart sections were stained with antibody against Nrf2 (ab137500,1:100) in a humidified box at 4°C overnight and followed by incubation with fluorescein-labeled secondary antibody for 1 h at 37°C. The cell nuclei were stained with DAPI for 5 min. All images were captured with a fluorescence microscope or scanner by Caseviewer software (3D Histech).

### Measurement of CK-MB, LDH, ALT, Creatinine in Serum and MDA, SOD, GSH, and GSH- Px in Tissues

The CK-MB, LDH, ALT, creatinine levels in serum were detected using the commercial kits (Changchun Huili Biotech Co., Ltd.) according to the instructions. In addition, the heart tissues were placed in cold saline (1: 10, w/v) and then homogenized with a homogenizer machine. Next, the supernatant was obtained through centrifuging at 3000 r/min to detect the MDA, SOD, GSH, and GSH- Px levels in heart tissues according to the instructions (Jiancheng Biotech Co. Ltd, China).

### Histopathologic Assay

Heart tissues were fixed in 10% formalin and embedded in paraffin, and then the sections were stained with hematoxylin-eosin (H&E) solution. Finally, images of the stained sections were obtained by Olympus microscope or scanner (3D Histech). The images were graded by the degree of myocardial necrosis and inflammatory cell infiltration according to the following standards: grade 0, normal; grade 1, lesion not exceeding 25%; grade 2, lesion between 25–50%; grade 3, lesion between 50–75%; grade 4, lesion exceeding 75% (Kanda et al., 2004). Six sections of one heart were graded by an experienced pathologist, who was blinded to the study design. The mean score of the six sections was recorded as the final cardiac pathology score.

### Western Blot

The total protein samples from the cells and heart tissues were homogenized using RIPA lysis buffer containing protease and phosphatase inhibitors (Beyotime, Shang Hai, China). The protein concentrations of the samples were determined using a BCA Protein Assay Kit. After determining the contents, the proteins were separated by SDS-PAGE (8–12.5%) and then transferred to PVDF membranes (Millipore, Massachusetts, United States). After being blocked with 5% skim milk for 2 h at room temperature, the membranes were incubated with primary antibodies overnight at 4°C. The following antibodies were used. Hmox1 (10701-1-AP, 1:1000), Bax (50599-2-Ig, 1:2000), Bcl-2 (12789-1-AP, 1:1000), lamin B1 (12987-1-AP, 1:1000) were purchased from proteintech. Antibody against cleaved caspase-3 (9661, 1:1000) was from CST. Antibody against Nrf2 (ab137500, 1:1000) was purchased from Abcam. Then the bands were incubated with secondary antibody for 1 h at room temperature. The protein bands on the membranes were detected using an enhanced chemiluminescence system (WBKLS0500; Millipore, Darmstadt, Germany). Intensity values of the relative protein levels were normalized to β-actin (*in vitro*) or α-tubulin (*in vivo*).

### Real-Time PCR Analysis

Total RNA was extracted with RNAiso Plus (Takara, Kusatsu, Japan) from NRCMs and heart tissues. cDNA was synthesized using Evo M-MLV RT Kit (AG, Hunan, China). Quantitative real-time polymerase chain reaction (RT-PCR) was performed with Hieff^®^ qPCR SYBR Green Master Mix (Yeasen, Shang Hai, China) on a QuantStudio 3 Real-Time PCR System (Applied Biosisytems, Waltham, MA, United States). The sequences of primers are shown in Supplementary Table S1.

### Transfection of Nrf2-siRNA

siRNAs were synthesized by Ribobio (Guangzhou, China). We transfected Nrf2-siRNA or negative control siRNA using Rfect siRNA/miRNA Transfection Reagent (Baidai biotechnology, Changzhou, China) according to the manufacturer’s instructions when cells reached 40–50%. The transfection efficiency was evaluated by Western blot. The used siRNA sequences are as follows: SiRNA1: CAAACAGAATGGACCTAAA;SiRNA2:GCAAGAAGCCAGATACAAA;SiRNA3:GGATGAAGAGACCGGAGAA.

### Data Analysis

The data are expressed as the mean ± standard deviation (SD). Statistical analysis was performed with GraphPad Prism 5.0 software (San Diego, CA, United States). It was performed with one-way analysis of variance (ANOVA) followed by Tukey’s posthoc test when comparing multiple groups, whereas differences within two groups were evaluated by Student’s *t*-test. Survival analysis was performed using the Kaplan–Meier method. Statistical significance was defined as *p* < 0.05.

## Results

### The Protective Effects of DMF Against DOX-Induced NRCMs Damage

**FIGURE 1:**
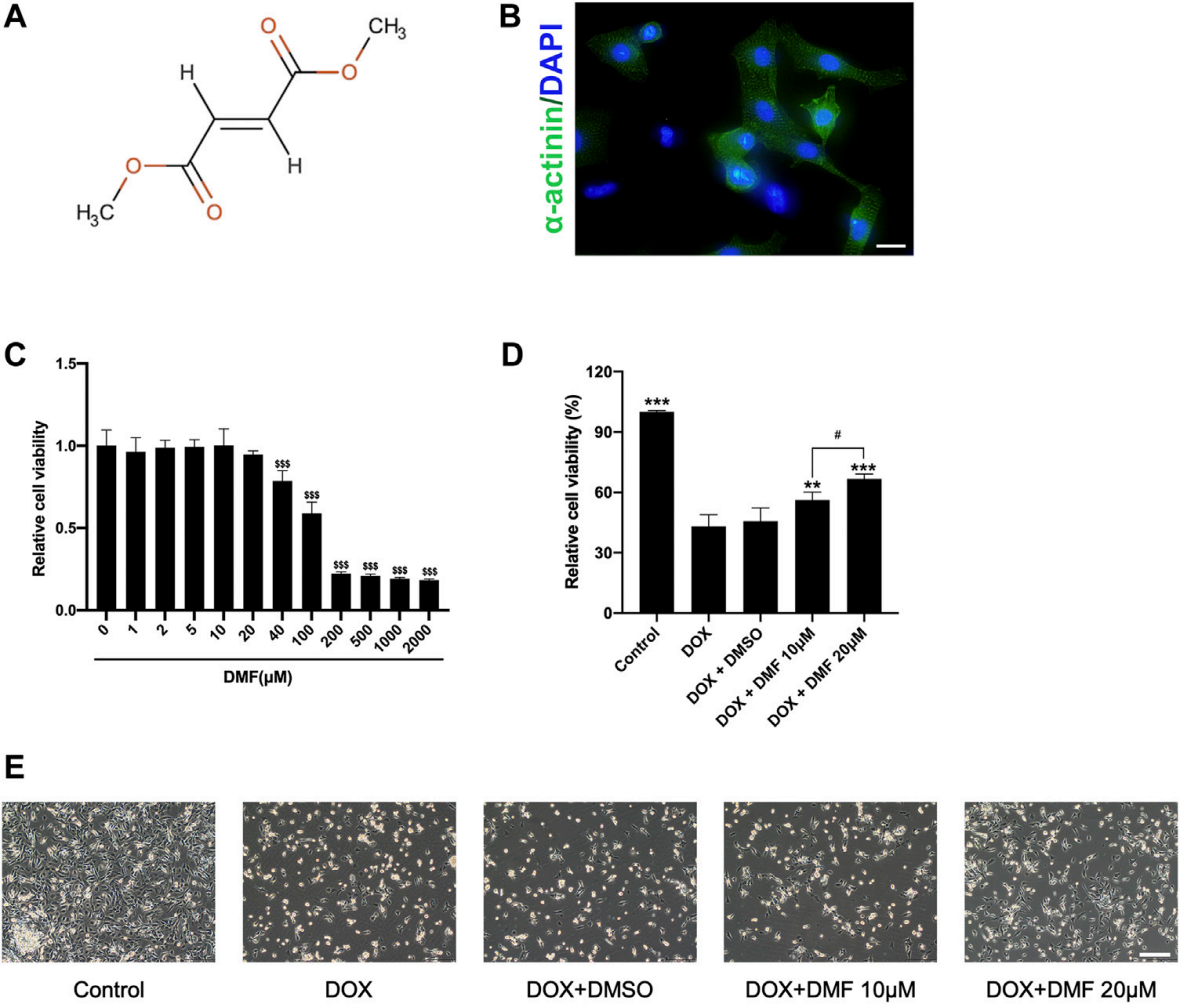
DMF alleviates NRCMs damage against DOX. **(A)** Chemical structure of dimethyl fumarate. **(B)** Immunofluorescence of α-actinin in NRCMs (scale bar = 20 μm). **(C)** Cell viability of NRCMs with different concentrations of DMF (*n* = 4). **(D)** Changes in cell viability (*n* = 4). **(E)** Changes in cellular morphology (*n* = 4, scale bar = 50 μm). Data were presented as the mean ± SD.^$$$^ *p* < 0.001, compared with the control group, ***p < 0.001, compared with the DOX group. ^#^ *p* < 0.05, compared with DOX + DMF 10 μM group.

Firstly, NRCMs purity was identified by immunofluorescence for α-actinin, a cardiomyocyte-specific marker (Figure 1B). Then the concentration-dependently cytotoxicity of DMF was evaluated by CCK8 assay. Compared with the control group, the viabilities of NRCMs were significantly reduced over 40 μM DMF for 24 h. As a result, we conducted concentrations of 10 and 20 μM DMF in this research (Figure 1C). NRCMs were pre-treated with DMF for 4 h, then treated with 5 μM DOX for 48 h. Compared to the control group, DOX treatment caused a significant decrease in cell viability and impaired cell morphology. However, DMF could concentration-dependently improve the viability of NRCMs and cell morphology damage compared with the DOX group (Figures 1D,E). Furthermore, the solvent control of 0.1% DMSO showed no effects on the cells.

### The Protective Effects of DMF Against DOX-Induced Cardiac Damage *In Vivo*

**FIGURE 2:**
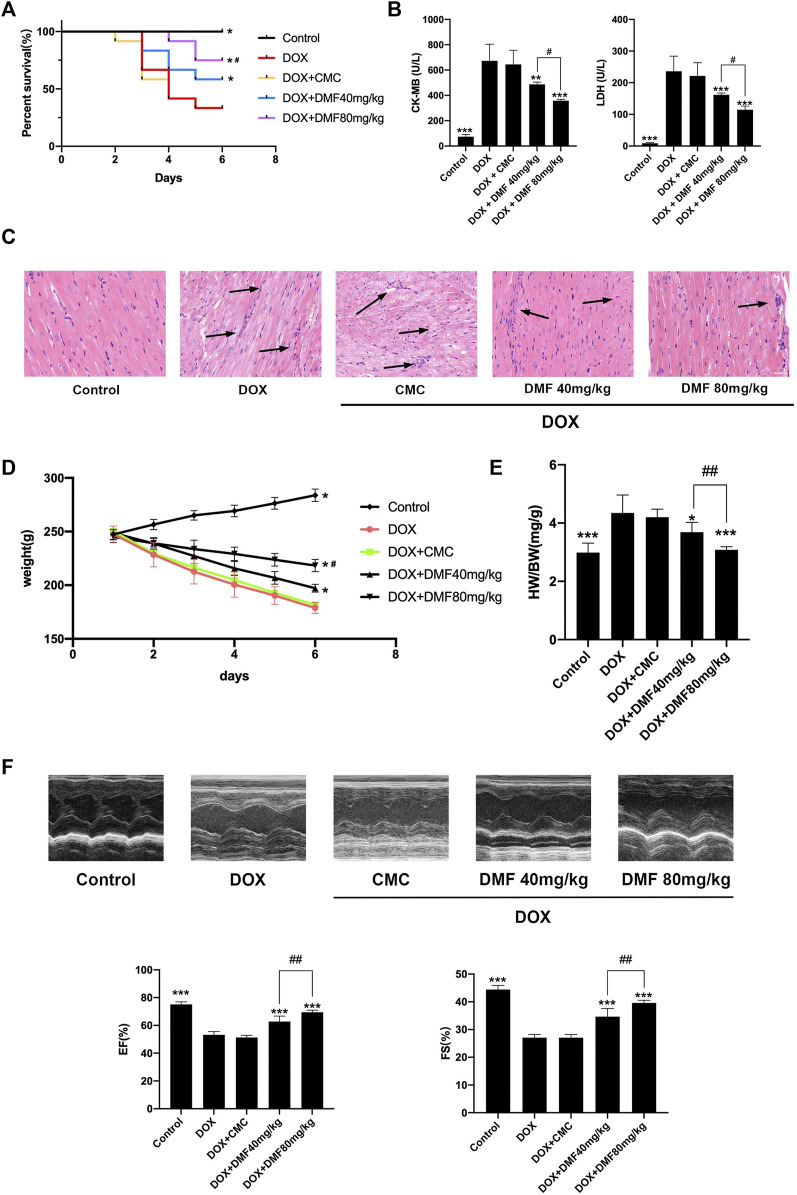
DMF alleviated DOX-induced cardiac injury. **(A)** Effects of DMF on Kaplan-Meier survival curves (*n* = 12). **(B)** Changes in serum levels of CK-MB and LDH (*n* = 6). **(C)** Representative H&E staining of hearts (Black arrows indicate the cardiac injury sites, *n* = 6, scale bar = 20 μm) **(D)** Changes in the body weights (*n* = 6). **(E)** Changes in HW/BW (mg/g) (*n* = 6). **(F)** Representative M-mode echocardiograms and quantitative analysis of LVEF and FS(*n* = 5). Data were presented as the mean ± SD. *p < 0.05, **p < 0.01, ***p < 0.001, compared with DOX group. ^#^ *p* < 0.05, ^##^ *p* < 0.01, compared with DOX + DMF 40 mg/kg group. HW: heart weight; BW: body weight.

Treating rats with DOX resulted in about 67% mortality compared with the control group. However, pre-treatment with a 40 mg/kg DMF showed a mortality of 42% and 25% with a dose of 80 mg/kg (Figure 2A). The serum CK-MB and LDH levels in DOX groups increased compared with control groups. However, DMF dose-dependently decreased the serum CK-MB and LDH levels (Figure 2B). The pathology of DOX-induced myocardial injury mainly includes sarcoplasmic reticulum expansion, cardiomyocyte edema, fiber rupture, and massive inflammatory cell infiltration (Ferrans et al., 1997). Consistent with previous studies, DOX caused apparent myocardial tissue disturbance, necrosis, and massive inflammatory cell infiltration, as well as higher cardiac pathology scores, which were dose-dependently alleviated by DMF. The control solvents of 0.8% CMC showed no effects on animals (Figure 2C, Supplementary Figure S1).

Considering heart failure is the most severe side effect after DOX treatment, we evaluated indicators related to heart failure. We found that the body weight was dramatically reduced after DOX treatment and can be reversed with an increasing dose of DMF (Figure 2D). The heart weight/body weight ratio, a simple indicator of heart failure, was increased in the DOX group and can be dose-dependently reversed by DMF (Figure 2E). Furthermore, the echocardiography confirmed the cardiac function improvement in DMF after DOX treatment (Figure 2F).

### DMF Inhibited Cardiac Oxidative Stress Caused by DOX

**FIGURE 3:**
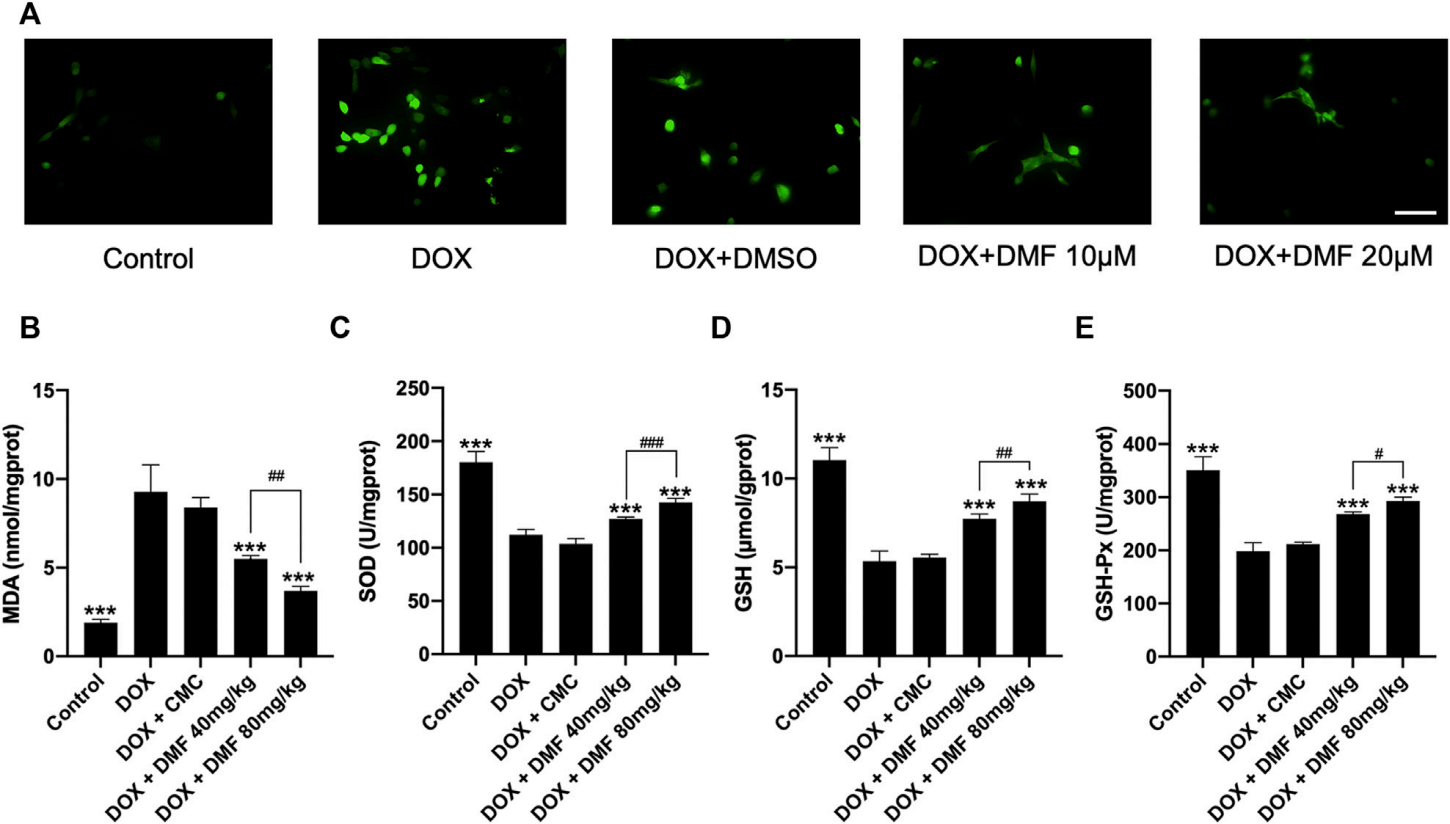
DMF alleviated oxidative stress *in vitro* and *in vivo*. **(A)** Effects of DMF on cellular ROS level in NRCMs treated by DOX (*n* = 5, scale bar = 50 μm). **(B–E)** Effects of DMF on the levels of MDA, SOD, GSH, and GSH-Px in hearts after DOX treatment (*n* = 6). Data are presented as the mean ± SD. ***p < 0.001, compared with DOX group. ^#^ *p* < 0.05, ^##^ *p* < 0.01, ^###^ *p* < 0.001 compared with DOX + DMF 40 mg/kg.

Given that oxidative stress is crucial in DOX-induced cardiac damage, we tested the oxidative stress levels *in vitro* and *in vivo*. The intracellular ROS level in NRCMs in the DOX group was remarkably increased compared with the control group and was decreased by DMF (Figure 3A). Then we measured some indicators representing tissue oxidative stress levels in heart tissue. MDA levels were elevated in DOX groups and were significantly decreased by DMF. DMF restored the levels of SOD, GSH, and GSH-Px, which were downregulated in the DOX group (Figures 3B–E). These results indicated that DMF could alleviate DOX-induced oxidative stress *in vitro* and *vivo*.

### DMF Alleviated DOX-Induced Cardiac Apoptosis

**FIGURE 4:**
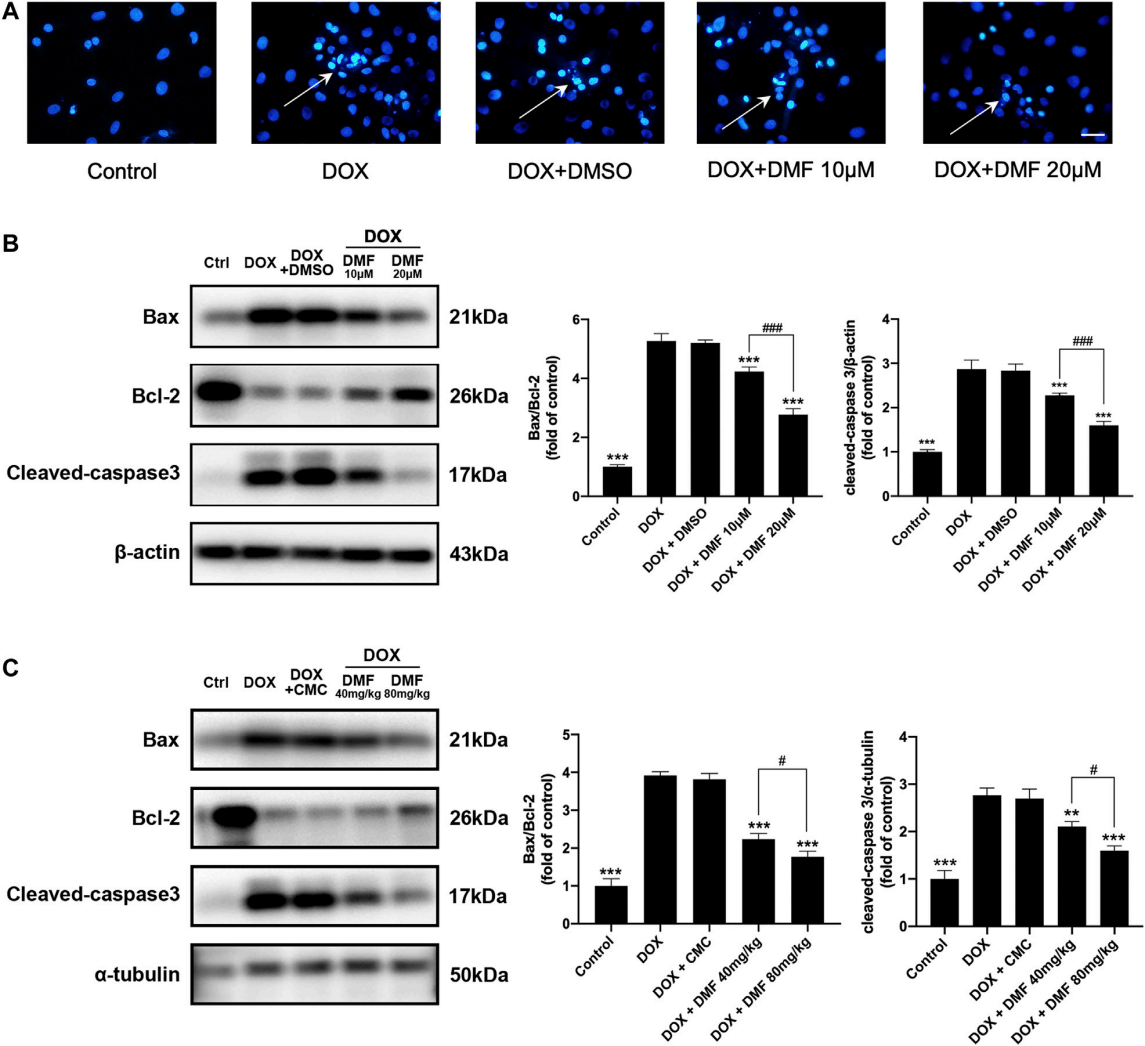
DMF inhibited DOX-induced cardiac apoptosis. **(A)** Hoechst 33258 staining of NRCMs after DOX (White arrows indicate apoptotic cells, *n* = 5, scale bar = 50 μm). **(B)** Representative WB images and quantitative analysis of Bax/Bcl-2 ratio and cleaved caspase-3 in NRCMs (*n* = 5). **(C)** Representative WB images and quantitative analysis of Bax/Bcl-2 ratio and cleaved caspase-3 in rats (*n* = 5). All data were expressed by mean ± SD. **p < 0.01, ***p < 0.001, compared with DOX group. ^#^ *p* < 0.05, ^###^ *p* < 0.001 compared with DOX + DMF 40 mg/kg or DOX + DMF 10 μM.

Apoptosis-related cardiomyocyte death is the leading cause of heart failure after DOX treatment, so we analyzed apoptosis indicators *in vitro* and *vivo*. The apoptotic cells were notably increased in the DOX group compared with the control group. However, treatment with DMF reduced the number of apoptotic cells (Figure 4A). Then, we evaluated the expression of apoptosis-related proteins. We found that administration of DOX increased the ratio of Bax/Bcl-2 and dramatically elevated the levels of cleaved caspase-3. In contrast, treatment with DMF notably decreased the Bax/Bcl-2 ratio and the levels of cleaved caspase-3 (Figure 4B). Consistent with results *in vitro*, DMF significantly reduced the ratio of Bax/Bcl-2 and the levels of cleaved caspase-3 in rats after DOX treatment (Figure 4C).

### DMF Promotes Nrf2 Pathway Signaling

**FIGURE 5:**
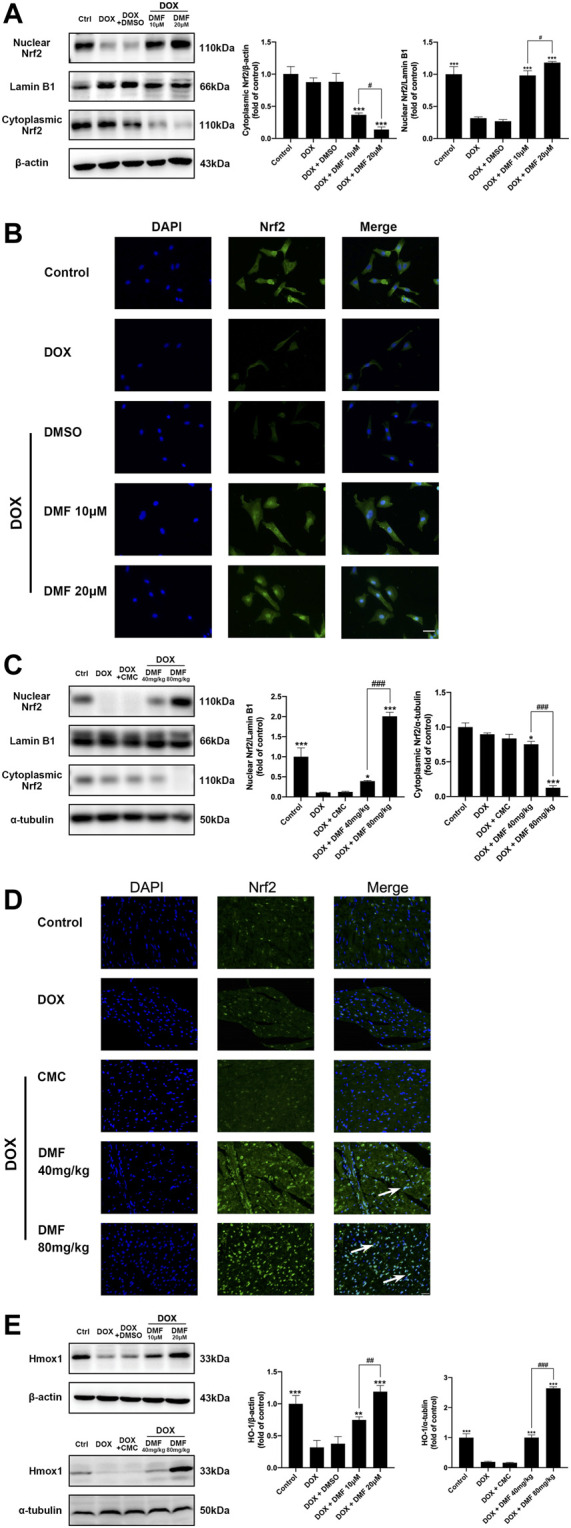
Effect of DMF on Nrf2 signaling in NRCMs and rats after DOX treatment. **(A)** Representative WB images and quantitative analysis of nuclear and cytoplasmic Nrf2 expression in NRCMs. β-actin and Lamin B1 were served as cytoplasmic or nuclear internal controls, respectively (*n* = 5). **(B)** Location of Nrf2 in NRCMs using Immunofluorescence (*n* = 5, scale bar = 25 μm). **(C)** Representative WB images and quantitative analysis of nuclear and cytoplasmic Nrf2 expression in rats. α-tubulin and Lamin B1 were served as cytoplasmic or nuclear internal controls, respectively, (*n* = 5). **(D)** Location of Nrf2 in rats using immunofluorescence (White arrows indicate the nuclear entry of Nrf2, *n* = 5, scale bar = 20 μm). **(E)** Representative WB images and quantitative analysis of Hmox1 expression in NRCMs and rats (*n* = 5). Data are mean ± SD. *p < 0.05, **p < 0.01, ***p < 0.001, compared with DOX group. ^#^ *p* < 0.05, ^##^ *p* < 0.01, ^###^ *p* < 0.001 compared with DOX + DMF 10 μM or DOX + DMF 40 mg/kg.

Nrf2 remains inactive in the cytoplasm under normal physiological conditions and enters the nucleus when activated. The expression levels of nuclear and cytoplasmic Nrf2 in NRCMs and heart tissues were assessed by western blotting. The results indicated that the expression levels of nuclear Nrf2 expression were significantly decreased, and the levels of cytoplasmic Nrf2 were slightly reduced in the DOX group. However, DMF significantly increased the expression levels of nuclear Nrf2 and decreased cytoplasmic Nrf2 levels (Figures 5A,C). These results suggested that DOX inhibited the entry of Nrf2 into the nucleus, which can be reversed by DMF. Then we further evaluated the Nrf2 translocation by immunofluorescence assay. Results *in vivo* confirmed that DOX significantly inhibited Nrf2 translocation, which can be dose-dependent reversed by DMF (Figures 5B,D). Hmox1 is one of the most powerful proteins in the Nrf2 pathway to defeat oxidative stress. We then tested its expression *in vitro* and *in vivo*. Results showed that DOX could significantly inhibit the expression of Hmox1, and DMF can dose-dependent increase it (Figure 5E). And other Nrf2 downstream antioxidant genes (NQO1, GCLC) showed similar changes (Supplementary Figure S2). These results indicated that DMF could activate the Nrf2 pathway by promoting Nrf2 transporting to the nucleus.

### DMF Exerts Protective Effects Through Nrf2

**FIGURE 6:**
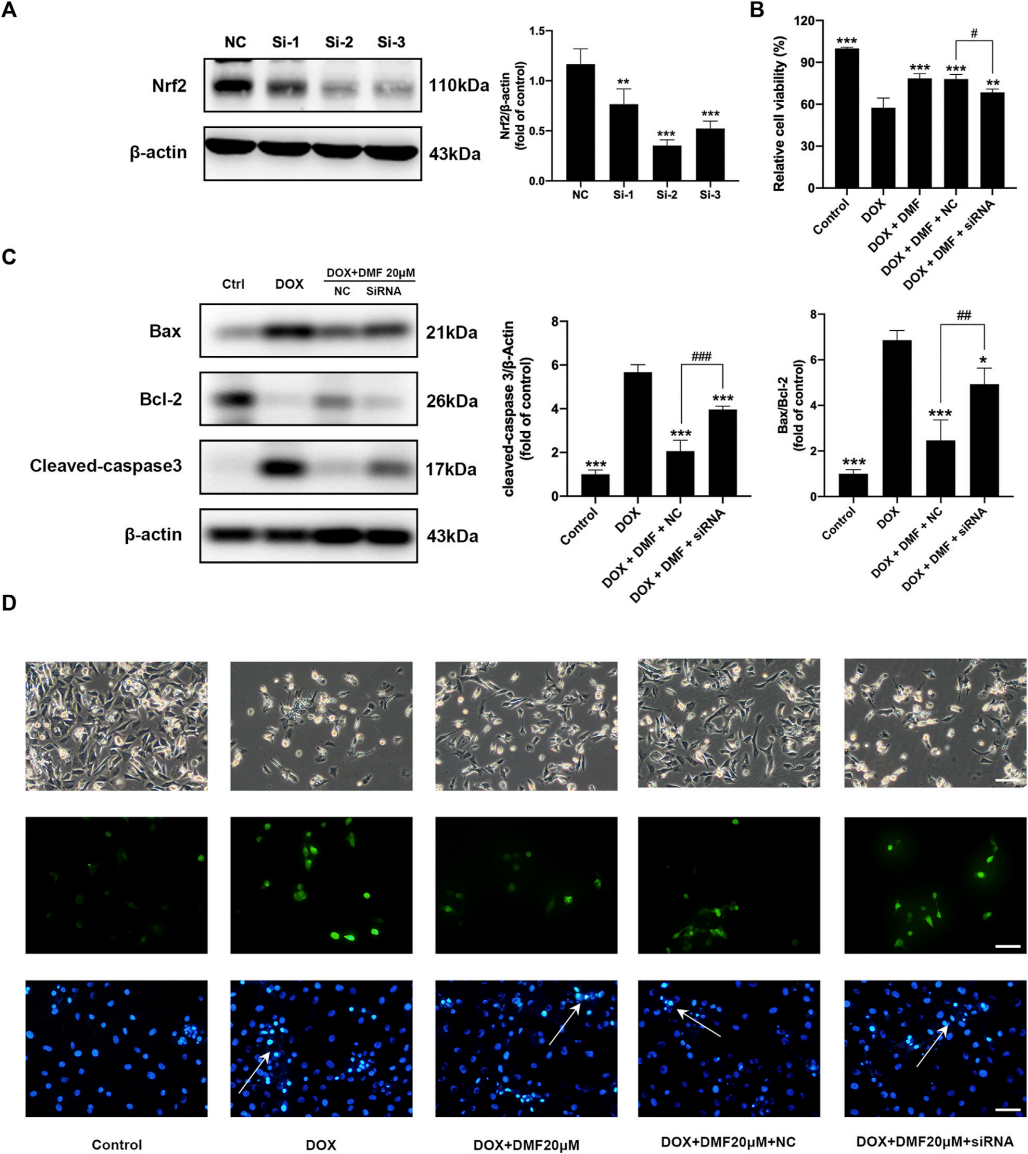
Nrf2 silencing reversed the protective effects of DMF on DOX-induced NRCMs injury. **(A)** Representative WB images and quantitative analysis Nrf2 expression in NRCMs after siRNA transfection (*n* = 5). **(B)** Changes in cell viability in NRCMs (*n* = 4). **(C)** Representative WB images and quantitative analysis Bax/Bcl-2 ratio and cleaved caspase-3 expression in NRCMs (*n* = 5). **(D)** Changes in cell morphology (*n* = 5), ROS levels (*n* = 5), and Hoechst 33258 staining in NRCMs (White arrows indicate apoptotic cells, *n* = 5, scale bar = 50 μm). All data were expressed by mean ± SD. *p < 0.05, **p < 0.01, ***p < 0.001, compared with DOX or NC group. ^#^ *p* < 0.05, ^##^ *p* < 0.01, ^###^ *p* < 0.001 compared with DOX + DMF + NC group.

To confirm the role of Nrf2 in the protective effects of DMF, the Nrf2-siRNA was conducted. Compared with the NC group, the protein levels of Nrf2 in NRCMs were most downregulated in the siRNA-2 group, which was used for further *in vitro* experiments (Figure 6A). Transfection with Nrf2-siRNA reversed the cell viability improvements by DMF (Figure 6B). Moreover, the cell morphology damage, ROS level, apoptosis levels, and antioxidant genes expression were reserved after Nrf2 silencing (Figures 6C,D, Supplementary Figure S3). These results suggested that DMF protected against DOX through the Nrf2 pathway.

### DMF Does Not Interfere With the Antitumor Ability of DOX

**FIGURE 7:**
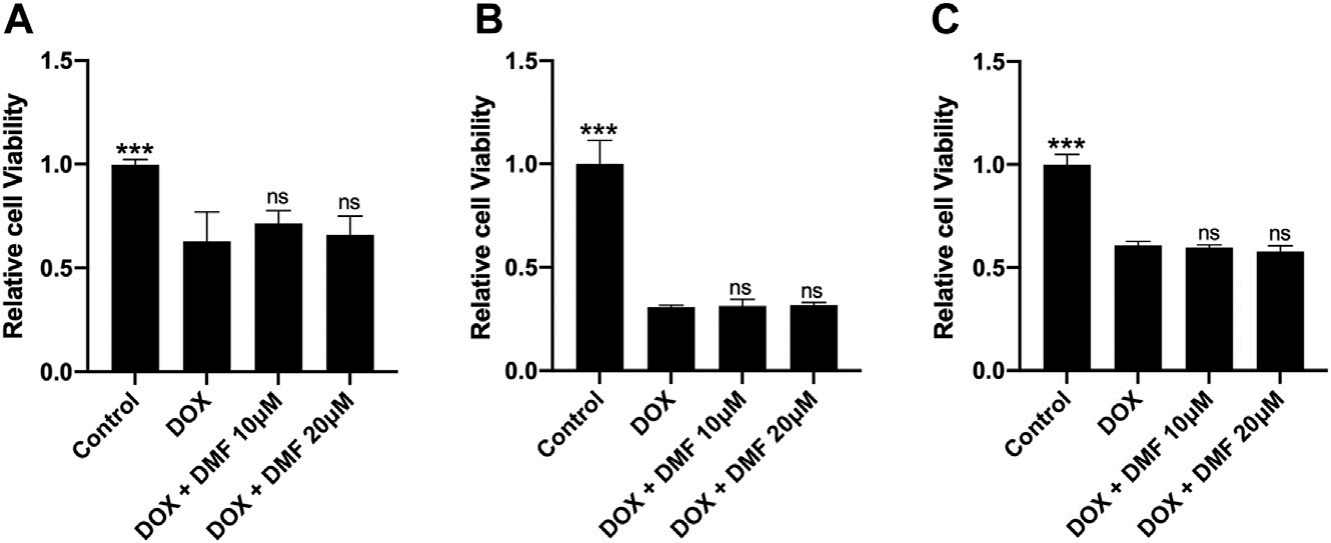
Effects of DMF on antitumor efficacy of DOX in some tumor cell lines. **(A)** Cell viability of SH-SY5Y cell line (*n* = 4). **(B)** Cell viability of CT26. WT colon cancer cell line (*n* = 4). **(C)** Cell viability of renal cell adenocarcinoma (Renca) cell line (*n* = 4). All data were expressed by mean ± SD. ***p < 0.05 compared with DOX groups, ns (not significant).

It was reported that Nrf2 activation could promote tumor cell proliferation and lead to chemotherapy resistance (Singh et al., 2016). So, we tested the effects of DMF on tumor cell viability after DOX treatment. Results showed that DMF exerted no protective effects on SHSY-5Y, RenCa, CT26. WT tumor cell lines after DOX (Figure 7).

## Discussion

DOX is a powerful and effective chemotherapeutic drug for solid and hematogenous cancer since 1969 (Damiani et al., 2016). However, the clinical utility of this drug is limited for its severe cardiotoxicity when it exceeds the cumulative dosage of 400–700 mg/m^2^ for adults and 300 mg/m^2^ for children (Li and Hill, 2014). The cardiotoxicity mainly includes arrhythmia and congestive heart failure (Mitry and Edwards, 2016). However, the precise mechanism of DOX-induced cardiotoxicity is still elusive. Many mechanisms contribute to DOX cardiotoxicity, such as ROS overload, iron metabolism disorder, mitochondrial dysfunction, calcium dysregulation, inflammatory cascade, endothelial dysfunction, and apoptosis. Among those, oxidative stress plays a central role in DOX-induced cardiotoxicity. The abundant mitochondria within cardiomyocytes and inadequate endogenous antioxidant mechanism suggest that the heart is more susceptible to oxidative stress damage (Goffart et al., 2004).

After DOX administration, Massive ROS is produced during the redox cycle at complex I of the electron transport chain, leading to ATP synthesis disorder. In general, ROS-related enzymes within the mitochondria can reduce DOX to semiquinone, which can be readily reacted with oxygen to generate superoxide anions. Besides, DOX binds to free iron to generate iron-DOX complex, which can react with oxygen and catalyze Fenton reaction to produce massive ROS (Gutteridge, 1984; Simunek et al., 2009). The generated ROS then reacts with mitochondrial biomolecules (including lipids, proteins, and nucleic acids), disturbing mitochondrial function (Eder and Arriaga, 2006). Meanwhile, cardiomyocytes can reduce oxidative stress by some endogenous critical antioxidant enzymes, including SOD, GSH, GSH-Px. Among them, GSH and GSH-Px can catalyze the reduction of other peroxides, and SOD can reduce O^2-^ to low toxic H_2_O_2_ (Sugden and Clerk, 2006).

Nrf2 is a crucial intracellular oxidative stress regulator (Kopacz et al., 2020). Under oxidative stress, Nrf2 was depolymerized from Keap1 and translocated into the nucleus to activate various antioxidant genes (Hur and Gray, 2011; Kim et al., 2016). Nrf2 activation can alleviate arteriosclerosis, arrhythmia, and myocardial infarction by targeting ferroptosis, autophagy, programmed cell necrosis, and apoptosis (Chen, 2021). It was also reported that Nrf2 deficiency could aggravate cardiac injury after DOX treatment (Li et al., 2014).

As a powerful Nrf2 agonist, DMF would be speculated to alleviate cardiac oxidative stress caused by DOX. In the current study, DMF inhibited oxidative damage by downregulating levels of ROS *in vitro* and upregulating levels of SOD, GSH, GSH-Px *in vivo*. In addition, MDA, a significant ROS indicator, was significantly decreased by DMF. Then, the subcellular localization results of nrf2 suggested that DMF could promote nuclear translocation of Nrf2 and its downstream anti-oxidative gene (Hmox1) expression, which was inhibited by DOX. More importantly, the protective effects of DMF on oxidative stress could be eliminated by Nrf2 silencing. Collectively, these results indicated that DMF inhibited oxidative stress caused by DOX through the Nrf2 pathway.

Besides, DOX can activate MAPK, p38, and JNK pathways, which leads to apoptosis by disrupting Bcl-2, Bax, cleaved caspase-9, and cleaved caspase-3 balance (Xu et al., 2005). During apoptosis, caspase-3 is cleaved to an active form to degrade various functional proteins. Therefore, its activation is considered a sign of the inevitable stage of apoptosis (Crowley and Waterhouse, 2016). In our study, DOX activated the apoptosis pathway by increasing the Bax/Bcl-2 ratio and cleaved caspase-3 levels, which can be alleviated by DMF in a dose-dependent manner. Furthermore, Nrf2 silencing reversed the anti-apoptotic effects of DMF against DOX. These observations collectively indicate that DMF can attenuate apoptotic events caused by DOX through the Nrf2 pathway.

Recently, Fang reported that activating the Nrf2/Hmox1 pathway could aggravate cardiac ferroptosis in mice after DOX treatment by disturbing iron metabolism (Fang et al., 2019). However, the early Nrf2/Hmox1 activity (1 day after DOX treatment) and different DOX doses may not demonstrate the whole role of the Nrf2 pathway. Besides, ferroptosis is just one of various cell death types in cardiomyocytes induced by DOX. And DMF was reported to inhibit ferroptosis in multiple disease models by activating the Nrf2 pathway (Qiu et al., 2020; Zhang et al., 2020; Yan et al., 2021; Yang et al., 2021). Therefore, we need to further explore the relationship between the Nrf2/Hmox1 axis and ferroptosis in the heart after DOX treatment.

In addition to the heart, other organs, such as the skeletal muscle, brain, liver, and kidney, are also susceptible to oxidative stress caused by DOX. And kidney and liver are known to be the major metabolism organs for multiple drugs. Thus morphological and functional changes in the liver and kidneys were also examined. The results showed that DMF could also dose-dependently alleviate liver and kidney impairment caused by DOX (Supplementary Figures S4, S5). Together, we speculated that DMF is relatively safe and well-tolerated.

It was reported that Nrf2 activation could promote carcinogenesis and drug resistance (Hammad et al., 2019; DeBlasi and DeNicola, 2020). In this study, we didn’t detect that DMF could interfere with the effects of DOX chemotherapy on three tumor cell lines. Besides, DMF has also been shown some anticancer abilities in serval cancers such as melanoma, breast cancer, colon cancer, and lung cancers by targeting Nrf2, NF-κB, ERK1/2, and miRNA pathway (Yamazoe et al., 2009; Xie et al., 2015; Kastrati et al., 2016). Moreover, several clinical trials have been conducted to test the antitumor effects of DMF (Saidu et al., 2019).

Gastrointestinal adverse events such as nausea, heartburn, vomiting, and diarrhea are common in patients taking DMF (Fox et al., 2012; Gold et al., 2012). In the DEFINE/CONFIRM trials, the incidence of gastrointestinal adverse events was approximately 40%, leading to treatment interruption in 4% of patients (Phillips et al., 2015). And those adverse reactions were also seen in patients treated with DOX (Hesketh, 2008). Therefore, we need to closely monitor gastrointestinal reactions and give appropriate management once the two drugs are used in combination.

However, we admitted that this study has some limitations. We only selected two concentrations of DMF for the *in vitro* studies and two doses of DMF for the *in vivo* study, which may not fully demonstrate the pharmacological effects of this drug. Moreover, DMF was also reported to regulate immune cell activity, inflammation, and metabolism in various pathological models (Kornberg et al., 2018; Zhao et al., 2020). We cannot rule out that DMF may exert protective effects against DOX other than the Nrf2 pathway, which requires further exploration.

## Conclusion

**FIGURE 8:**
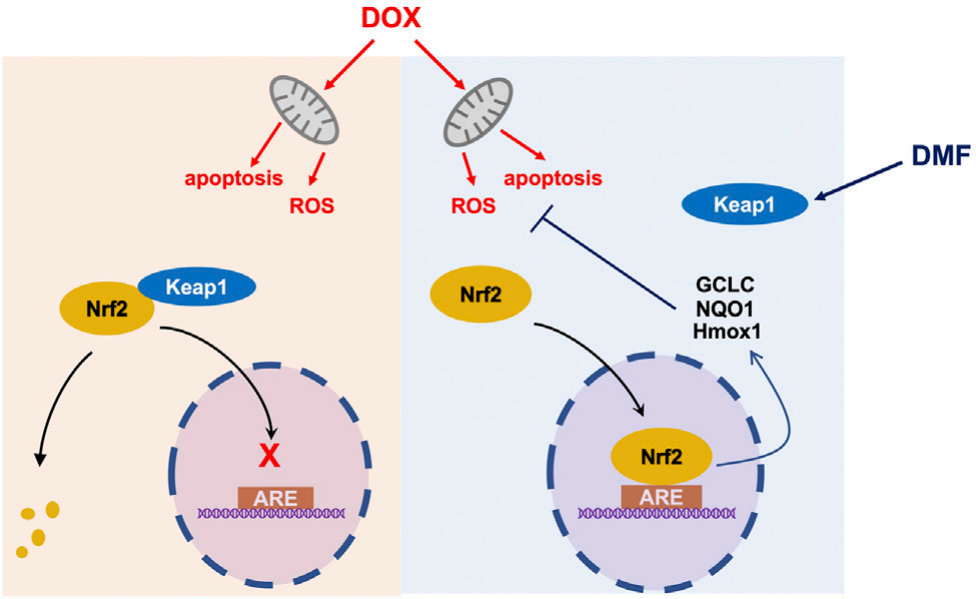
A schematic diagram of protection of DMF against DOX-induced cardiotoxicity through Nrf2 pathway. ARE Antioxidant Response Element.

In conclusion, our data showed that the FDA-approved drug DMF notably alleviated DOX-caused cardiac injury by activating the Nrf2 pathway without interfering with the chemotherapy effect of DOX, which should be developed as a promising candidate for patients suffering from DOX-related cardiotoxicity (Figure 8).

## Data Availability

The original contributions presented in the study are included in the article/Supplementary Materials, further inquiries can be directed to the corresponding author.
