# Supplementary material for: Dimethyl Fumarate Ameliorates Doxorubicin-Induced Cardiotoxicity By Activating the Nrf2 Pathway
Source: Front Pharmacol. 2022 Apr 26;13:872057. doi: 10.3389/fphar.2022.872057 (PMC9089305; doi:10.3389/fphar.2022.872057)
Supplement: Supplementary file 2 [file DataSheet1.docx]

Supplementary Material

1. Supplementary Figures

#
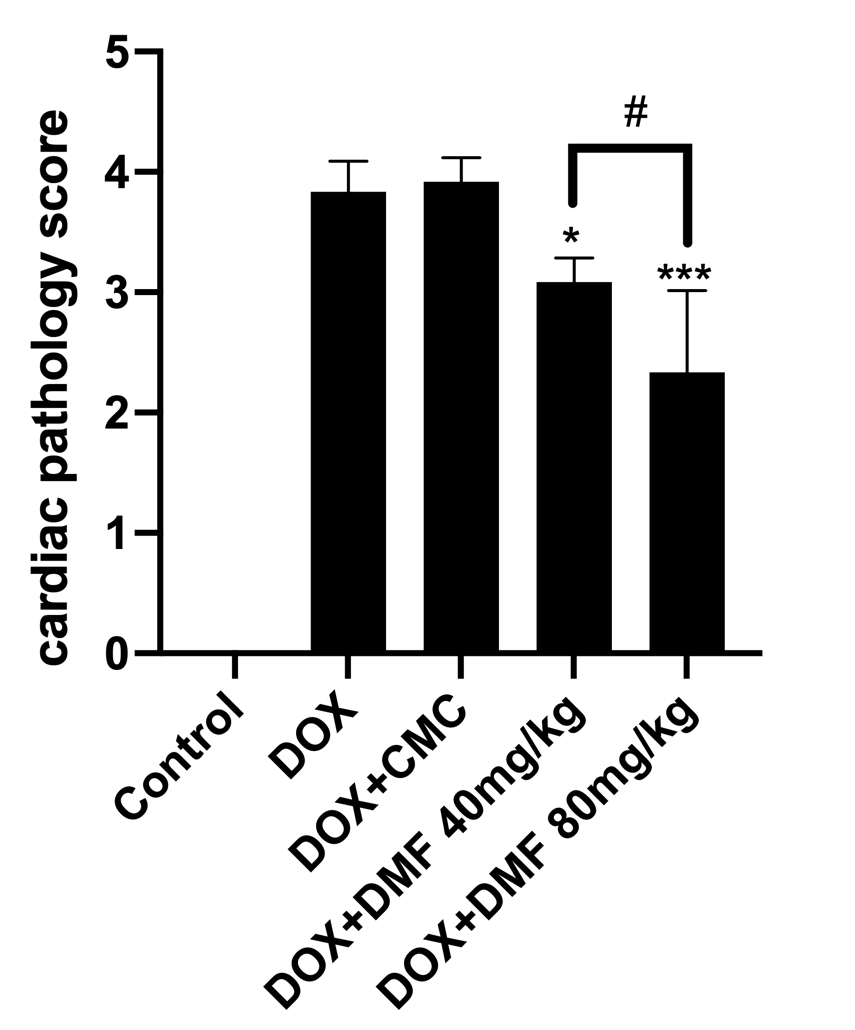


**Figure S1.** Effects of DMF on cardiac pathology score in rats. * p < 0.05, *** p < 0.001, compared with DOX group. ^#^p< 0.05 compared with DOX+DMF 40mg/kg group.

**Figure S2**. Effects of DMF on cardiac NOQ1 and GCLC mRNA expression. (A) Relative mRNA change in NRCMs. (B) Relative mRNA changes in rats. ** p < 0.01, *** p < 0.001, compared with DOX group. ^###^p< 0.001compared with DOX+DMSO or DOX+DMF 40mg/kg group.


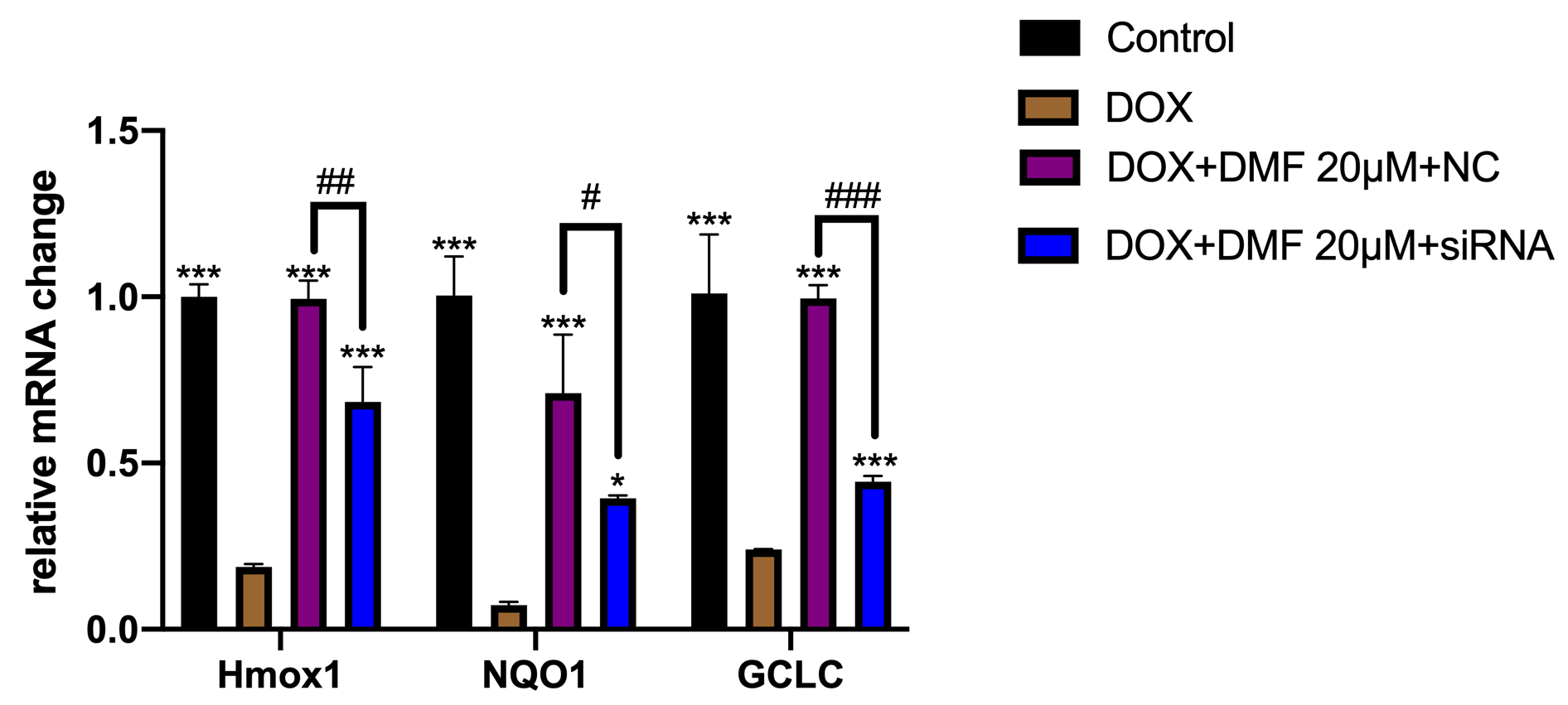


**Figure S3.** Effects of Nrf2 silencing on Hmox1, NQO1, GCLC expression. *** p < 0.001, compared with DOX group, ^#^ p < 0.05, ^###^ p < 0.001, compared with DOX+DMF 20μM+siRNA group.

#
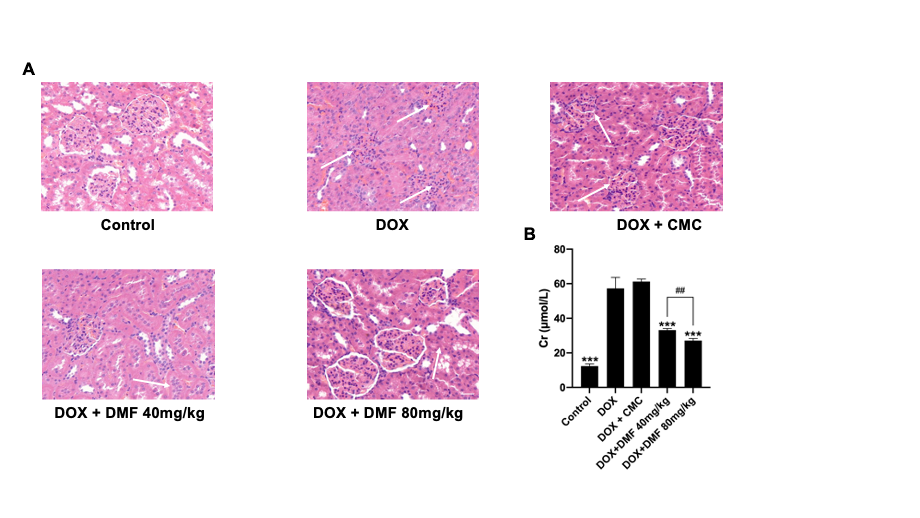


**Figure S4.** Effects of DMF on DOX-induced nephrotoxicity in rats. **(A)** Representative H&E staining of the kidney (White arrows indicate the kidney injury sites, n=6, scale bar = 50μm). **(B)** Changes in serum Cr levels (n=6). *** p < 0.001, compared with DOX group. ^##^p< 0.01compared with DOX+DMF 40mg/kg.


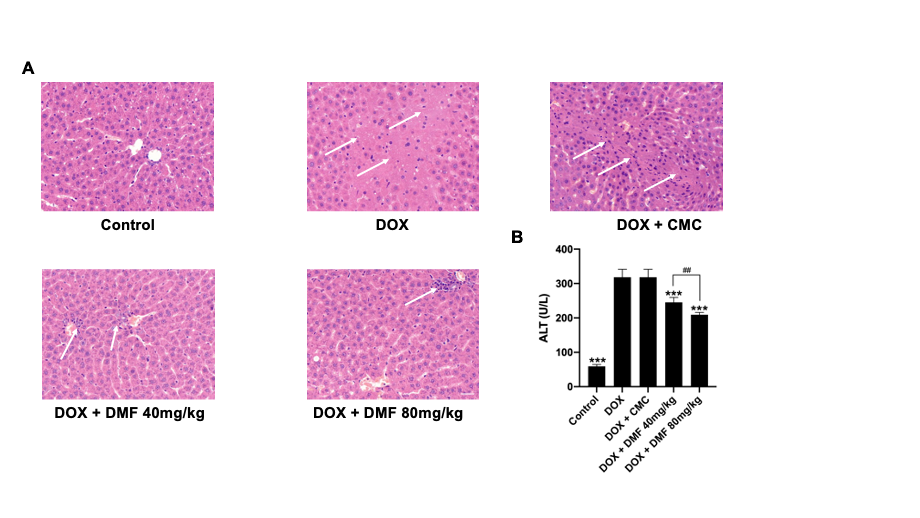
 **Figure S5.** Effects of DMF on DOX-induced hepatotoxicity in rats. **(A)** Representative H&E staining of the kidney (White arrows indicate the liver injury sites, n=6, scale bar = 50μm). **(B)** Changes in serum ALT levels (n=6). *** p < 0.001, compared with DOX group. ^##^p< 0.01compared with DOX+DMF 40mg/kg.

**Supplementary Table 1.** Table of primers sequences for Real-Time PCR

| Gene symbol | Forward primer | Reverse primer |
| --- | --- | --- |
| β-actin | ACCCGCGAGTACAACCTTCT | ATACCCACCATCACACCCTGG |
| Hmox1 | CAGGGAAGGCTTTAAGCTGGT | GTGGGGCATAGACTGGGTTC |
| NQO1 | CTGGCCAATTCAGAGTGGCAT | GAGTGGTGACTCCTCCCAGA |
| GCLC | CCGACCAATGGAGGTACAGT | TGTAAGACGGCATCTCGCTC |
